# Barriers and facilitators to programmatic mass drug administration in persistent schistosomiasis hotspot communities: An ethnographic study along Lake Albert, midwestern Uganda

**DOI:** 10.1371/journal.pntd.0012002

**Published:** 2024-12-13

**Authors:** Paskari Odoi, Stella Neema, Birgitte J. Vennervald, Edridah M. Tukahebwa, Shona Wilson

**Affiliations:** 1 Department of Sociology and Anthropology, School of Social Sciences, Makerere University, Kampala, Uganda; 2 Department of Veterinary and Animal Sciences, Faculty of Health and Medical Sciences, University of Copenhagen, Frederiksberg C, Denmark; 3 Vector Control Division, Ministry of Health, Kampala, Uganda; 4 Department of Pathology, University of Cambridge, Tennis Court Road, Cambridge, United Kingdom; James Cook University, AUSTRALIA

## Abstract

**Background:**

The WHO Neglected Tropical Disease Roadmap for 2021–2030 includes the goal of eliminating schistosomiasis as a public health problem in all endemic countries. Despite heightened efforts since 2012, critical action is still required in addressing barriers to Mass Drug Administration, the primary control method. This includes improvement in adherence by the populations in persistent schistosomiasis hotspots. One such hotspot is the shoreline of Lake Albert, Uganda, where schistosomiasis control is provided to school-aged children and adults. An overemphasis on regular treatment, without comprehensively addressing factors that result in low uptake of treatment in these high-risk populations is likely to impact the elimination of schistosomiasis as a public health problem.

**Methods:**

An ethnographic study using in-depth interviews, key informant interviews, focus group discussions and participant observation was conducted at two study sites along Lake Albert. Thematic content analysis was used during data analysis.

**Results:**

The study revealed that the size, taste and smell of the drug, along with its side-effects; poor community integration and occupational behaviour resulting in non-mobilisation; and unfounded rumours and beliefs remain reasons for persistent low uptake of praziquantel by some. Conversely, lived experience of improved health through participation and knowledge of the dangers of the disease if not treated, facilitated treatment uptake. Positive attitudes to localised sensitisation by community drug distributors show social influence facilitates crucial knowledge attainment. Treatment uptake is further facilitated by the delivery of the drug at no cost at home. Crucially, for the majority of participants the facilitating factors were found to outweigh the inhibitory factors related to the drug’s side effects.

**Conclusion:**

We recommend a good community engagement strategy that provides continuous education and sensitisation, with improved recruitment and training provision for Community Drug Distributors to facilitate programme reach to groups with current poor engagement.

## Author summary

Over the last two decades, in the Lake Albert region, Uganda, there have been a number of interventions targeted at schistosomiasis by grass root structures, and district and national level actors; but despite this the Lake Albertine districts remain a highly endemic region for schistosomiasis. In recognition of this persistent schistosomiasis, we examined the factors that inhibit or facilitate adherence to mass drug administration (MDA) using an ethnographic approach. Lived experience of improved health through participation and knowledge of the dangers of the disease if not treated, facilitated treatment uptake. Localised social influence was crucial in gaining knowledge that facilitated uptake. Key were positive attitudes to sensitisation conducted by community drug distributors; whilst uptake of treatment by children was reportedly heavily influenced by their mothers’ positive attitudes to treatment. The drug itself, praziquantel, was described as “very strong” and “effective” because of the relief from symptoms. There are, however, a number of people, both children and adults, who fail to adhere to MDA. Therefore, we recommend continuous education and sensitisation, alongside increased number and training of Community drug distributors/village health team members; with continued motivation for them as they are vital in enabling treatment uptake.

## Introduction

Schistosomiasis, caused by trematode worms of the genus *Schistosoma*, is one of the neglected tropical diseases (NTDs); a group of infections that disproportionately affect low-income populations. It remains a major public health problem in tropical and subtropical regions with an estimated 220 million people infected [1,2]. Transmission occurs through contact with water that has been contaminated with faeces or urine containing parasite eggs, and in which intermediate snail hosts breed. The urogenital or gastrointestinal tracts are impacted depending on the infecting species. Infection is associated with non-specific morbidities such as anaemia, growth retardation, and impairment of cognitive development, thus can lead to reduced work capacity; whilst long-term exposure to infection is associated with specific severe morbidities that can be fatal [3]. In sub-Saharan Africa, schistosomiasis is common, particularly in low—income areas without access to safe drinking water and adequate sanitation. At least 90% of those in need of schistosomiasis treatment are living in Africa [4].

Interventions to control schistosomiasis have varied over time and space. Early national control programmes, such as the Sudanese Blue Nile Health Project (started in 1979) and the Egyptian national programme (started in 1988), used a combination of chemotherapy, snail control, provision of clean water and sanitation, and infrastructural development [5,6]. Such multi-approach control programmes are now the exception rather than the rule [7], though the latest edition of the WHO roadmap for control of NTDs emphasises the need to return to multi-approach programmes [8]. Instead, due to the combined low cost, good safety record and high efficacy of the anti-schistosomal drug praziquantel (PZQ), preventative chemotherapy (PC) through mass drug administration (MDA) on a population scale became the mainstay of internationally funded national schistosomiasis control programmes. The WHO originally set a 2025 target for these PC programmes of schistosomiasis morbidity control (defined as <5% of school-aged children (SAC) having a heavy infection), followed by eradication as a public health burden where possible [8]. This has since been revised in the latest Roadmap for NTD control to elimination of schistosomiasis as a public health problem (defined as <1% of SAC having a heavy infection), in all endemic countries by 2030 [1]. However, schistosomiasis elimination efforts have rarely succeeded and only in relatively isolated situations [7]. Elimination requires extensive (and by extension expensive) integrated approaches, and when not feasible, the tools available have to be optimal to gain the greatest outcomes [9]. The elements that result in optimal tools are often not appreciated, hence the need for medical anthropological studies to improve understanding of programme limitations.

PC interventions involve a variety of elements relating to the target population, the drug to be used and the organisations implementing the intervention. These include population sensitisation, drug procurement and distribution, reporting accuracy and varying involvement of international partners, national government Ministries of Health and Education, and local government and community structures. The interactions of these elements cut across different spheres of influence (from international to local), resulting in non-linear effects on outcomes and a wide range of circumstances may have an impact on the results. In addition, several operational strategies for drug delivery can be adopted depending on the endemicity and national policies. These include variation in platform (school- or community-based), target groups (SAC or SAC alongside adults) and frequency (year or biennially). Given the complexity in PC programme design and implementation, identifying the barriers and facilitators that affect programmes’ ability to meet recommended targets is difficult.

Despite, the complexity, since its internationally supported inception in 2003, MDA using PZQ has led to substantial country-level success in attainment towards the original 2025 goals [10–12]. However, regardless of several years of well-implemented MDA coverage, variation in transmission, at times as focal as an individual village, can result in failure to substantially decrease prevalence and mean intensity of schistosomiasis infections at the micro-geographical level [13,14]. Precision mapping or research studies are required to detect these village-to-village differences and precise characterisation of a “hotspot” needs to be applied to understand why some villages fail to decline in their infection status. Only with this understanding can determination of whether enhanced MDA efforts alone will suffice or whether additional control measures will be required in individual areas [14].

In Uganda, MDA began in 2003 through the support of the Schistosomiasis Control Initiative (SCI; now Unlimit Health), and 400,000 PZQ treatments were distributed that year [15–17]. Subsequently, treatment coverage increased each following year with 1.8 million individuals treated by 2007 [18], resulting in significant reductions in *S*. *mansoni* prevalence, intensity, and associated morbidity [12,19]. Other countries employing MDA have reported similar successes [20]. As a result of these findings, WHO’s strategy for disease control in some African nations, including Uganda, has been updated to include the interruption of transmission by 2030 [21]. In common with other countries Uganda has areas of continued high schistosomiasis transmission or hotspots; and contributing to this continued high transmission could be coverage rates of the programme in these areas. Social factors are likely amongst those that play an important role in MDA uptake. An individual’s positioning in the community’s social network, trust in the community drug distributors (CDDs) for health advice [22], and engagement by the community leaders [23] have all been noted amongst facilitating factors. On the other hand, poor leadership, insufficient social engagement, lack of genuine community participation, low motivation by the CDDs and their commitment to schistosomiasis control [23], and the side effects associated with PZQ [24,25] are reported inhibitors of treatment uptake. To be able to avert the barriers while promoting facilitating factors in these persistent schistosomiasis hotspots, an understanding of what the local barriers and facilitators are and what causes them is required. The aim of the current paper is to investigate these barriers and facilitators of MDA, specifically in persistent hotspots along Lake Albert, Uganda.

## Materials and methods

### Ethics statement

The study protocol was approved by Makerere University School of Social Sciences Research Ethics Committee (MAKSSREC REF 04.19.283) and Uganda National Council for Science and Technology (UNCST REF No SS 5048). Data collection was conducted while respecting the confidentiality of the study participants. Participants were informed of confidentiality procedures as part of the consent process. Consent forms for each type of instrument were administered before the commencement of the interviews. The consent forms were translated into Alur and Lunyoro and written consent was obtained from the participants.

### Study design

The study employed an applied ethnographic study design with a blend of phenomenology approaches to capture the lived experiences of adults. By lived experience, we mean a representation and understanding of research participant’s human experiences, choices, and options and how those factors influence one’s perception of knowledge. An applied ethnographic study design uses ethnographic methods to address specific practical problems or questions in real-world settings [26].

### Study area and setting

The study was conducted in the western sub-counties of Kigorobya, Buseruka and Kabaale, Hoima District, which are located along the shores of Lake Albert. Hoima District is in the mid-western region of Uganda, with Hoima Municipality, the seat of the District Headquarters, around 200 km from Kampala using the direct Kampala–Hoima Road. It shares borders with Masindi and Buliisa Districts in the north, Kyankwazi District in the east, and Ntoroko, Kakumiiro, and Kagadi Districts in the south. To the west, Hoima District stretches to the national border with the Democratic Republic of Congo, located within Lake Albert. The district has a total area of 5735.3km^2^, a land area of 3612.17km^2^, and includes Lake Albert and other water bodies amounting to 2123.13km^2^of water [27]. For occupation, the majority of the people in areas above the Lake Albert basin escarpment (Kigorobya and Buseruka sub-counties) are agriculturalists with emphasis on food crop production, while the majority of those who reside near the shores of lake Albert (Kabaale subcounty) are fishermen and fisherwomen [27,28]. However, the recent discovery of oil in Kabaale sub-county, has led some to change their occupation to business and industrial work.

### Selection criteria and study participants

The study primarily targeted community members in and around Kaiso and Buhirigi Primary Schools, the participatory schools in the wider FibroScHot project (www.fibroschot.eu), in which this anthropology study is integrated. These community members included men, women, youths, and older adults with no upper age restriction. These study participants mainly speak Alur and Lunyoro, the two most frequently spoken local languages in Kaiso and Buhirigi. Other participants included Key Informants at the district, sub-county, and village levels. Participants were purposively selected. During observations and transect walks in the villages, several people were identified as key informants, often because of their position in the community. Their inclusion in the KIIs and IDIs depended on their interest in the study, schistosomiasis and/or health research and roles in the community. In addition, the fisherfolks were considered leaders of their different small groups, or were named key persons because of either their age and duration of stay in the community or involvement in fishing, a high-risk occupation for schistosomiasis. The FGDs participants were identified through assistance from the local council leadership and local FibroScHot Project Coordinators. The participants were selected based on knowledge, experience, and interest in the study with the aim of gaining a wide range of perspectives. Purposive sampling was used to select the participants. Stratification criteria were used to create clusters based on age (18 years and above), location (Kaiso and Buhirigi), gender (male and female), from which participants with homogenous characteristics were drawn.

#### Methods of data collection

The study utilised a rapid ethnographic approach with triangulation of unstructured participant observations, KIIs, IDIs and FGD, conducted using guides. Through this process, a picture of the communities’ receptiveness to programmatic MDA was built up and weaknesses, dislikes and any waning of willingness for themselves or their children to be involved identified. Two social science trained research assistants, one male and one female, who both spoke the two main local languages (Lunyoro and Alur) and a PhD student, spent six weeks in the study area. Prior to implementation of the study, 4-days of training was conducted to reinforce the data collectors’ capacity to collect data using qualitative methods. The training included research ethics, confidentiality and best practices in interviewing techniques and encompassed: 1) an overview of the study protocol, 2) research ethics, including consent taking, 3) a review of tools (FGD, KII and IDI guides) and 4) role plays. The data collectors participated in practical exercises to familiarise themselves with the instrument(s) that they would use in the field and a pre-test was conducted to refine the tools. The data collection and transcription within the communities was conducted November to December 2019. A total of 12 KII, 60 IDIs and 24 FGD were originally planned. Transcripts were reviewed by the study team after each day of activity and if data saturation of salient factors was deemed to have been reached [29], then the process of interviewing and conducting FGD was terminated.

#### Key informant interviews

KIIs were conducted at the district, sub-county, and village levels. These included local government officials and community leaders (civil, social, political, religious, opinion). In total 12 key informants were interviewed as planned.

#### In-depth interviews

In-depth interviews were conducted with purposively selected community members (men, women, youths, and older adults with no upper age restriction) who had experienced schistosomiasis either directly or indirectly through family or friends, in order to understand their perceptions and lived experiences (phenomenology) and coping strategies. During observations and transect walks in the villages, several people were identified as IDI participants through assistance from the local council leadership, local FibroScHot Project Coordinators, and Village Health Teams (VHTs). During this process, participants were screened by confirming their socio-demographic characteristics and whether they had had schistosomiasis or had a family member who had ever had schistosomiasis. A youth may be defined in several contexts. According to the United Nations, a youth is a person aged between 15 and 24 years. According to the African Union, a youth is a person aged between 15 and 35 years. In Uganda, a youth is a person aged 18 to 30 years [30]. For the purpose of our study, we utilised the Ugandan definition of youth. A total of 52 IDIs out of the planned 60 were conducted due to data saturation having been met.

#### Focus group discussions

FGDs were conducted with community members comprising of 6 to 7 participants per group. Group categories included gender and age (youth or non-youth) and were organised separately. A total of 14 FGDs out of the planned 24 were conducted due to data saturation having been met. In all, 97 people participated in the 14 FGDs.

#### Participant observations

Unstructured observations were conducted at the landing sites to ascertain the people’s activities such as water collection, bathing or working on fishing equipment that would expose them to schistosomiasis infection. Events were observed on the spot and recorded by use of recorders, cameras and/or field notes [31].

### Data management and analysis

Data was analysed using a thematic content analysis approach conducted on a rolling basis from transcription to result extraction. All interviews were recorded digitally, transcribed and typed by the research assistants. Notes collected during the interviews (formal and informal) including non-participant observation data, were expanded every evening by the data collectors. On return from the field, these notes, mostly in the form of jottings in paraphrases, were expanded into fully developed sentences and paragraphs to make sense of what was written. Recordings from the FGDs, IDIs and KIIs were transcribed verbatim by the research assistants and quality checked by the principal investigator. The transcripts were entered in Nvivo qualitative analysis package (Version 12) for coding and analysis. A 4-step analysis method of transcripts was used [32], in which: 1) each individual transcript was read to gain familiarisation with the responses of the study participants, and to note major opinions and attitudes; 2) similar themes and sub-themes were grouped into codes; 3) inductive evolution of the code structure to reflect participants’ experiences and voices and finally, after coding; 4) development of the categories and themes and their interrelationships. The data from the different tools were analysed independently and then triangulated to enhance rigour. Finally, the themes were reviewed together to ensure inter-rater reliability [33,34]. The results were categorised and interpreted in relation to the objectives of the study and quotes used extensively to further explain and provide evidence for the emergent themes [35]. Quotes are attributed to the tool by which the quote was obtained, the participant’s gender, their age group (for IDIs and FGDs only) as defined as 18–24 and 25–30 for youths and then in 5-year age groups between 31 and 60 years of age, with all those over 60 being grouped together, and the village (IDIs and FGDs only) in which they are resident. Key identifiers of participant’s roles have been removed for KII responses.

## Results

### Socio-demographic characteristics of the participants

#### In-depth interviews

The socio-demographic characteristics of IDI participants are shown in S1 Table. By age over half of the participants interviewed in IDIs were youths (54.3%); 56.6% were male; 65% were married; just below a half (47%) reported having had some secondary education (Senior 1 to Senior 4), and a further 45% had some primary education; 45 (85%) had previous knowledge about schistosomiasis, and one reported currently having schistosomiasis.

#### Focus group discussions

Of the fourteen FGDs conducted, eight comprised of male participants while six FGDs comprised of female participants. The age of participants ranged from the 18–24 year-old age group to the 51–55 year-old age group. Most of the participants, especially in Kaiso, were fishermen and fishmongers, whereas in Buhirigi many were engaged in farming and a few in fishing. A few of participants had never been to school, but the majority had at least some primary education, followed by secondary education. Many reported having previously been ill with schistosomiasis, more so in Kaiso. The socio-demographic characteristics by FGD are available in S2 Table.

#### Key informant interviews

The 12 key informants included district administrative officials and health officials, sub-county chiefs and local councillors.

## Facilitators of mass drug adminstation

### Participation in mass drug administration

#### Good sensitization

Participants commonly reported to have been sensitized on the dangers of the disease (schistosomiasis) (‘*bilazia’* by the Alur and ‘e*mpuka’* by the Banyoro) and on PZQ (‘*baya*’ as referred to by the Alur) as a drug that is effective in treating schistosomiasis. By living at the lake shores, they knew they were exposed to and at risk of getting the schistosomiasis. They were also aware of the MDA programme and its purpose. This compelled them to participate in MDA as they wanted to get cured and live in good health. One male FGD participant shared that:

*“Some people in the community participated in MDA because they were very much aware that living along the shores of Lake Albert, which is the breeding ground for the organism, puts them at risk of getting schistosomiasis”* ***(FGD—Males, 23–30 years old, Kaiso).***

#### Delivery within their community

A further identified facilitating factor was that MDA took place within their community. They reported that the medicine was distributed by the people they knew and trusted and that the CDDs and VHTs brought the medicine to their households, free of charge. Furthermore, people participated because they were encouraged and had been registered by the CDDs/VHTs who followed them up in their homes.

*“They also participate because the community drug distributors are people who come from within the community and they know them very well”* ***(KII–Local Leader 1).***

#### Lived experience of schistosomiasis and the benefits of treatment

There were those who had seen that people who had used the medicine were healed. They reported that having seen and shared experiences with recovered cases encouraged them to participate. Others participated because they took health issues seriously as their own responsibility. Those who suspected they had been infected or had symptoms of schistosomiasis participated as the severity of the disease instigated them to take action.

*“Because some people want to make good use of any opportunity to treat schistosomiasis, if they could be having it”* ***(IDI–Female, 21–25 years old, Kaiso)***.

*“Some participated because they have experienced schistosomiasis and they are aware how terrible it is*. *So*, *they want to avoid catching it again”* ***(IDI–Male*, *26–30 years old*, *Kaiso)*.**

#### Other facilitators of participation

By gender, more women actively participated than men because they wanted to set a good example to the children and the whole family. They also made sure that their children participate in MDA and took their drugs. For the school-going children, they participated because it was organised in schools and it was perceived to be compulsory.

*“Women were the ones who participated most because they were ever active and eager to participate in everything. Men do not participate because they always claim they have no time–they are ever busy”* ***(IDI–Male, 41–45 years old, Buhirigi).***

Respondents commonly reported that every member of the community who valued health and knew the benefits and effectiveness of the medicine, participated in MDA programmes. Specific groups of people were pointed out, like the children who were targeted at school and those who were supported by their mothers; it was stressed that mothers participated in the MDA as responsible parents.

#### Facilitating perceptions of the drug praziquantel

When asked to talk more specifically about PZQ, the drug for schistosomiasis, all categories of respondents reported that the medicine was “very strong” and “effective”. All those who had previously had schistosomiasis reported that the drug was effective in treating it, providing relief from the symptoms they had suffered. They expressed that they got better, were cured or healed after using it. They also liked that it worked fast.

*“The medicine is very good. When I started taking the medicine, I started improving very fast and the symptoms began to disappear* ***” (IDI—Male, 41–45 years old, Kaiso).***

*“I like its effectiveness*. *Today I am alive and speaking to you because the medicine helped me”* ***(IDI—Male*, *26–30 years old*, *Kaiso****)*.

More of what people liked about the medicine was that it helped or made them discover they had schistosomiasis, as they were not aware of any symptoms prior to treatment, but they were evident through improvement in health after taking the medicine; whilst for others it helped them confirm they did not have symptomatic schistosomiasis.

In probing further about what the participants liked about PZQ, the theme of local availability and the importance of communication about the disease through CDDs and VHTs that was identified during questioning about the MDA programme more generally, was again apparent. The participants reported they liked the medicine because it was free of charge and was brought to their home. Hence no costs incurred.

*“They like its ability to cure the disease, and moreover it is brought for them at their homes”* ***(KII- Local Leader 1).***

*“They continue taking the drug because their community drug distributors together with the village health team (VHTs) sensitised the community members on the dangers of the disease and the benefits of the drug”* ***(KII–Local Leader 2)*.**

There were those who were happy it was available through government services. Furthermore, the service providers were reported to have followed up their patients for adherence, which was appreciated, and they also liked the awareness they got on the dangers of the disease and the benefits of the medicine. This combined with the opportunity they had to share their experience with champion patients (i.e., people identified in the community to talk about the effectiveness of treatment) and survivors encouraged them in adherence.

*“They say they like it for the fact that it cures them. Most of the people after taking it tell you they now feel better than before”* ***(FGD–Males, 20–35 years, Buhirigi).***

There were also some “facilitating” misconceptions. The drug was reported to have treated unrelated ailments by killing other infections. It was also reported to have been liked because it prevented further schistosome infections.

#### Motivation to continue participation

A number of factors were reported to motivate people to continue with taking the drug in the future. First, there was desire for complete healing. Some people had hoped they would get well because they were seeing some improvement after starting on the treatment. Some in-depth interview participants reported they wanted to continue with the drug because they were aware of the continued risks of disease if they didn’t take the drug when still exposed to lake waters; and thus, exposure to the disease. Motivation was enhanced because they had seen and heard of the survivors’ testimonies. They also knew the dangers of the disease, that if not treated it could be fatal and they knew it had killed people in their community.

*“I continued taking the medicine because I found it of great help to my situation and also, I feared to contract the disease again because I still live in the same landing site with the threat of the disease”* ***(IDI—Male, 31–35 years old, Kaiso).***

## Barriers to mass drug administration

### Programmatic barriers to participation in mass drug administration

#### Poor mobilization

This was caused by a fishing method called ‘s*alacio*’ which is an Alur concept that refers to a fishing method where fishermen stay in the lake for quite a long time e.g., a month or more. As a result of this fishing behaviour, information may not have reached some people in the community as captured during one of the KII:

*“There are fishermen in this community who spend a long time in the lake fishing. Some of these men can be in the lake for weeks and even a month without returning home”* ***(KII- Local Health Worker).***

#### Ignorance

The finding of ignorance was common among more peripheral members of the community such as Congolese migrants; contrasting directly for those members of the community who are permanent residents who were often reached out to by the CDDs. This ignorance amongst the Congolese was attributed to the protracted insecurity in the Democratic Republic of Congo (DRC), resulting in a continuous movement the Uganda side of Lake Albert, where they settle on the landing sites. Most of the Congolese migrants were Congolese of Alur origin. Whenever there is relative peace in DRC, some of these migrants attempt to go back. Thus, the frequent movement of these migrants makes it hard for them to consistently adhere to MDA as captured during one of the IDIs:

*“Some people don’t want to participate in MDA in this community because they are not well informed about schistosomiasis and do not know about the intervention programme”* ***(IDI–Male, 26–30 years old, Kaiso).***

*“Some people especially the Congolese come and go back after some time because they do not know the importance of the medicine”* ***(KII—Local Leader 1)***.

However, most of the Congolese migrants in this study were new or recent arrivals in the study area, which partly accounted for their ignorance of the MDA.

#### Perception of wellness

Some people did not participate because they knew they were not infected or thought they were safe and not at risk of getting infected with schistosomiasis. The very old people did not participate because they thought it was useless as they were in their last days of life.

*“Some people are just defiant. Some older men especially, claim the disease is not a threat to them because they were born and raised drinking the lake water and they are still healthy”* ***(KII—Local Health Worker).***

#### Misconceptions

Perceptions of the impact on fertility were identified as an inhibiting theme, with rumours of reduced sexual desire, and some men reported not to have participated because they believed the drug made them infertile or made them impotent. Pregnant women were reported not to have participated because they believed the drug to cause abortion or were informed that it would affect them in some other way and were recommended not to participate:

*“Some people don’t want to take the medicine because of rumours that medicine was brought to reduce their sexual desire to control reproductive rate. This is common belief among some people here in this community”* ***(FGD -Males, 31–74 years old, Kaiso).***

*“It is because some women are pregnant so they are not supposed to take the medicine praziquantel”* ***(IDI—Female*, *26–30 years old*, *Kaiso)*.**

#### Non-programmatic barriers to participation in mass drug administration

Wider non-programme specific social factors were also found to have an impact. It was reported that political influence was one reason for non-participation with members of the opposition discouraging people to take the drug.

*“There is also political influence from opposition who persuade people not to take the drug saying that it is bad, with a fake aim of decampaigning and frustrating government programmes for their own political gain”* ***(KII—District Official).***

Other people did not participate due to religious affiliations and beliefs. These were religions which do not allow use of any medicines, except prayers. One of such group was the Triple six (666) who don’t take medicines as stated by one the IDI participant:

*“Here we have the religious sect called Triple Six (666). They don’t take the medicine because their religion does not allow them to take medicine of any kind”* ***(IDI—Male, 26–30 years old, Kaiso).***

Finally, other people were said to be careless with their lives, including drunken men who did not care about their lives, and hence did not participate.

*“Others don’t take because of ignorance and carelessness as you can see when you move through the village, there are some categories of people who generally don’t care about life”* ***(KII—District Official).***

#### Inhibiting perceptions of the drug praziquantel

When asked about factors that may inhibit participation in MDA, it was common for respondents to report that people feared the side effects of the drug (PZQ). They reported side effects that included diarrhoea, dizziness and nausea amongst others. The most common side effects presented were stomach pain/ache and diarrhoea. Other side effects included vomiting, body weakness, body rashes, dizziness, swelling of the body particularly the stomach, giving a bad odour/smell, loss of appetite, serious headache, itching of the body, and change in body/skin and hair colour and texture. These side effects were reported to clear with time. Further, the big size, taste and smell of the medicine were other reasons for non-participation. However, it was found that when the participants had lived experience of improved health after taking the drug, the side effects were not inhibitory:

*“People say the medicine is very strong and effective and one should take it when he/she is well prepared for it physically by eating enough and psychologically because of the side effects”* ***(KII–Local Health Worker).***

*“I have personally taken this medicine*. *It is good and it helped me, but it made me pass a lot of diarrhoea and I became weak” **(FGD—Males, 22–35 years old, Buhirigi).***

*“*Praziquantel *causes stomach ache*, *diarrhoea and vomiting*, *but that is how it eliminates the germs from the body”* ***(IDI—Female*, *26–30 years old*, *Kaiso)*.**

Despite the identified drug-related barriers, including misconceptions, participants commonly reported they were willing and had nothing to prevent them from taking their medicines.

*“Nothing can prevent me from taking the medicine because the benefits of the medicine are more than the side effects”* ***(IDI—Male, 26–30 years old, Kaiso).***

*“Following the threat the disease gave to my life*, *I don’t think anything can prevent me from taking the medicine”* ***(IDI—Male*, *>60 years old*, *Kaiso)*.**

## Discussion

The three high *S*. *mansoni* endemicity sub-counties of Buseruka, Kabaale and Kigorobya, are situated in Hoima District, Western Uganda which has a long Lake Albert shoreline. This geography, combined with the socio-ecological interactions between the lake shore communities and the lake itself, results in this area being one of the most significant hotspots of schistosomiasis transmission globally. To determine the influence of social factors in participation in the control programme, this study examined the barriers and facilitators of MDA within these communities.

As for the facilitating factors, study participants commonly reported that they had been sensitised on the dangers of the disease (schistosomiasis) and PZQ as a drug that is effective in treating schistosomiasis and they were aware of the MDA programme and its purpose. This compelled them to participate in MDA because they wanted to get cured and live in good health. We additionally found that those who suspected having been infected, or those with symptoms of schistosomiasis participated in treatment to a higher degree. Additionally, participants knew that receiving no treatment can lead to death, and therefore such fears compelled them to participate. Participants reported that they wanted to continue with the drug because they were aware of the risks when still exposed to lake waters and by extension to the disease; thus, the preventative rather than curative benefits of participation were also understood. Several studies from elsewhere in Uganda also reveal that the lived experience of the health benefits of treatment and of knowing others who have had improved health after taking PZQ, leads to MDA uptake [18,24,36]. At the same time, quantitative analysis has shown that knowledge of the disease is a positive predictor of treatment [37] and systematic review of the factors affecting the uptake of preventive chemotherapy treatment for schistosomiasis in sub-Saharan Africa also revealed that people participate in PC programmes as a result of their knowledge and awareness of the signs, effects, and transmission cycle of schistosomiasis [3]. Together these findings highlight that health communication regarding the disease is vital for strong uptake of PC.

People also participated in MDA programmes because they were encouraged and registered by the CDDs/VHTs who visit homes to carry out sensitisation and encourage participation. The significant role played by CDDs in sensitising the communities is in line with the findings of Corley et al. [38] and Krentel et al. [39], who highlight CDDs as a “valuable human resource” in the motivation and education of communities, promoting the sustainable uptake of NTD activities. Krentel et al. went on to recommend the recognition of CDD contribution by programme managers, whilst acknowledging that non-performance in volunteer roles can also inhibit programmatic achievements [39]. Fleming et al. [40] also cite positive and negative influence of these volunteers on programmatic outcomes in the Ugandan districts of Kamuli, Pallisa, Mukono, and Yumbe. For instance, positive influence was seen through their participation in sensitisation and mobilisation campaigns, treatment and writing of coverage reports based on their treatment register. On the other hand, lack of availability, capacity, acceptance, and, indeed, ownership of the programme by the CDDs led to negative outcomes. Similar findings have been reported from the other East African countries of Rwanda [41] and Tanzania [42]. Our participants further reported that it was not only that the CDDs/VHTs provided information, but that they knew the people who distributed the medicine and placed importance on this. In Kagadi and Ntoroko Districts on the southern shores of Lake Albert CDDs/VHTs are the primary health seeking point of contact for schistosomiasis, inferring trust is held in them [43], whilst in Mayuge District, Uganda a direct tie (close friendship) between CDDs and community members was associated with enhanced treatment uptake [22]. These findings resonate with those of our study; emphasising the need for CDDs to be picked from those well-connected within the community. The delivery of the drug at home by the CDDs, free of charge, as a facilitator was also a key finding.

When it comes to the inhibiting factors, we found out that participants commonly reported that people feared the nasty side effects of PZQ; a well reported barrier to the uptake of treatment [37,44,45]. In the Ugandan context, this barrier has been found both qualitatively [46] and quantitatively [37]. Unlike our study, which involved youth and adults, both these studies involved children and adults as their target respondents and so age-related influences cannot be ruled out as a reason for differences in the influence of side-effects on uptake. In particular, in our study, the benefits of treatment were found to outweigh the fear of these side effects for many participants, and in fact, many linked these side effects to the strength and effectiveness of the drug.

Although women are more likely to take part, worries during pregnancy can be an inhibitory factor. Pregnant women were reportedly not participating because they were worried that the drug caused abortion or affected them in some other way. They also reported that they were informed not to participate. WHO has indicated that pregnancy is often cited as a reason for non-compliance [47], potentially resulting from the ineligibility of pregnant and lactating women in the early years of the programme; a recommendation which has since been reversed for those beyond the first trimester of pregnancy [48]. Reproductive and sexual fears amongst men through beliefs of the drug making them either infertile or impotent were also reported. The same unfounded fear was noted in Mayuge District, Uganda [44] and Morogoro, Tanzania [49]. If such misconceptions are not addressed it can drastically limit the uptake of PC.

Some people were reported to be ignorant about the programme, especially the Congolese migrants. For a group such as these, there is a need for effective communication and community engagement to ensure compliance with MDA and other treatment measures [50]. We found that because of the protracted insecurity in the DRC, most of the Congolese migrants in this study were new or recent arrivals. Parker *et al* [24] previously reported a great deal of distrust and antipathy between some locals and migrants in Nebbi District, north-western Uganda, which presents additional communication and community engagement challenges. As a result, some community members miss out on health education and mobilisation, leading to a lack of or inadequate knowledge about MDA. Such lack of or inadequate knowledge among the migrant population has been reported in other studies, as highlighted by the systematic review of knowledge, attitudes, and practices related to schistosomiasis in sub-Saharan Africa [51]. Cross-border movement and its negative impact on programmes occurs across health spheres that use an MDA approach [52], so its negative influence is likely be seen in other public health programmes being implemented in these communities.

Another reason for poor mobilisation was information concerning MDA implementation not reaching some people in the community due to their economic behaviours, in particular a fishing method called ‘s*alacio*’ as practiced by some of the fishermen. The same finding was reported for fishing communities in Kagadi and Ntoroko Districts [43]. In a situation of this nature, Adriko et al. [37] in their study in Mayuge District, Uganda, suggest that improved educational campaigns and effective mobilization could increase health-seeking behaviour and improve community-wide MDA, even to such community members. A study by Coulibaly et al. [53] in Côte d’Ivoire supports these conclusions with findings that anytime was suitable for treatments if people were informed sufficiently in advance. Whilst in their systematic review, Torres Vitolas *et al* argue that there is a need to involve trusted local leaders with a reach to the sectors of communities missed during VHT and CDD mobilisation activities [54]. In response to their findings in Kagadi and Ntoroko Districts, Ayonlitho et al recommend strengthening the CDD/VHT structure to allow sustainable availability of PZQ for those who seek treatment outside of the control programme distribution window [43].

Some people did not participate because they knew they were not infected or they thought they were safe and not at risk of getting infected with schistosomiasis. Whilst the very old did not participate because they didn’t see the need as they have always lived in the area (and by extension with the disease). This overlaps with research from Zanzibar where people reported missing PZQ because they felt healthy [55] and is an example of interacting bio-social factors contributing to low drug coverages [37].

Concerning study limitations, the study did not coincide with the months for MDA, so corroboration through structured observation of how the benefits of treatment outweighed the fear of side effects was not possible. Secondly, we acknowledge that the duration spent on data collection (the six weeks of November to December) could have limited data collection from study participants who are subject to a migratory process. Equally important and related to migration, the study tools were only translated into two main local languages, Alur and Lunyoro, and interviews were conducted in English, Alur and Lunyoro. Thus, those who speak other minority languages present amongst these lakeshore communities may be missing amongst our participants due to a language barrier.

## Conclusion and recommendations

Our research findings indicate a range of inhibiting and facilitating factors in the adherence to MDA. The inhibiting factors include, among others, social factors that have a contextual consequence on uptake, such as religious and political influence and positioning within the community that can lead to ignorance of the programme; but also factors directly related to the intervention, notably the side effects, size, taste and smell of the drug. However, facilitating factors such as knowledge of the severity of the disease, and the fact that people take their health seriously have greatly facilitated praziquantel uptake. Equally important, the localised, no cost availability of the drug, and the sensitisation and drug delivery by known individuals, combined with lived experience, either their own or of others, were perceived to outweigh the negative factors related to the drug and its side effects. Additionally, there is strong evidence that the participants had a very good understanding about why the side effects occur and their transitory nature and for the majority side-effects were not inhibitory to uptake of treatment.

Arising from the study findings we propose that more needs to be done to counter the unfounded rumours about the drug, and to reach those not receiving messaging regarding MDA from the CDDs. The findings highlight a need for continuous education and sensitisation on schistosomiasis causes, prevention and treatment to the community members; particularly given that most of them are mobile workers with occupations conducted at the lake. A good community engagement plan or strategy is important and should include the use of expert patients/champions/survivors, alongside local leaders and health workers to advocate for the drug and to encourage uptake in the community. Involvement of all stakeholders: district, village, traditional and religious leaders alongside the CDDs and VHTs in mobilising people for participation will be crucial. Sensitisation, recruitment, training and motivation of drug distributors from within the communities is also key. Finally, treatment supporters such as patient’s relatives, community volunteers working with the programme, medical workers, and pharmacists within the community among others could help to assuage fears of side effects and counter unfounded rumours to ensure greater uptake.

## Supporting information

S1 Table Socio-demographic characteristics of the in-depth interview participants. Provided are the summary statistics for age, gender, educational level, marital status and reported *S*. *mansoni* infection status of participants.
(DOCX)

S2 Table Socio-demographic characteristics of the focus group discussion participants. Provided are the ranges in age, educational status and the gender of the participants for each group.
(DOCX)

## References

[1] World Health Organization. WHO guideline on control and elimination of human schistosomiasis. Geneva: World Health Organization; 2022. 35235279

[2] World Health Organization. Schistosomiasis: Fact Sheet. WHO; 2019. [cited 2024 Feb 15]. Available from: https://www.who.int/news-room/factsheets/detail/schistosomiasis

[3] Torres-Vitolas CA, Dhanani N, Fleming FM. Factors affecting the uptake of preventive chemotherapy treatment for schistosomiasis in Sub-Saharan Africa: A systematic review. PLoS neglected tropical diseases. 2021;15(1): e0009017. doi: 10.1371/journal.pntd.0009017 33465076 PMC7846123

[4] World Health Organization. Schistosomiasis: Fact Sheet [Internet]. WHO; 2023. Available from: https://www.who.int/news-room/factsheets/detail/schistosomiasis

[5] Fenwick A, Webster JP. Schistosomiasis: challenges for control, treatment and drug resistance. Curr Opin Infect Dis. 2006;19(6):577–582. doi: 10.1097/01.qco.0000247591.13671.6a 17075334

[6] el Gaddal AA. The Blue Nile Health Project: a comprehensive approach to the prevention and control of water-associated diseases in irrigated schemes of the Sudan. J Trop Med Hyg. 1985;88(2):47–56. 4032529

[7] Rollinson D, Knopp S, Levitz S, Stothard JR, Tchuem Tchuenté LA, Garba A, et al. Time to set the agenda for schistosomiasis elimination. Acta Trop. 2013;128(2):423–440. doi: 10.1016/j.actatropica.2012.04.013 22580511

[8] World Health Organization. Accelerating work to overcome the global impact of neglected tropical diseases: a roadmap for implementation. Geneva: WHO; 2012.

[9] Secor WE, Colley DG. When should the emphasis on schistosomiasis control move to elimination? Trop Med Infect Dis. 2018;3(3):85. doi: 10.3390/tropicalmed3030085 30274481 PMC6161309

[10] Deol AK, Fleming FM, Calvo-Urbano B, Walker M, Bucumi V, Gnandou I, et al. Schistosomiasis—Assessing progress toward the 2020 and 2025 global goals. N Engl J Med. 2019;381(26):2519–2528. doi: 10.1056/NEJMoa1812165 31881138 PMC6785807

[11] Andrade G, Bertsch DJ, Gazzinelli A, King CH. Decline in infection-related morbidities following drug-mediated reductions in the intensity of Schistosoma infection: a systematic review and meta-analysis. PLoS Negl Trop Dis. 2017;11(2):e0005372. doi: 10.1371/journal.pntd.0005372 28212414 PMC5333910

[12] Kabatereine NB, Brooker S, Koukounari A, Kazibwe F, Tukahebwa EM, Fleming FM, et al. Impact of a national helminth control programme on infection and morbidity in Ugandan schoolchildren. Bull World Health Organ. 2007;85(2):91–99. doi: 10.2471/blt.06.030353 17308729 PMC2174620

[13] Wiegand RE, Mwinzi PNM, Montgomery SP, Chan YL, Andiego K, Omedo M, et al. A persistent hotspot of Schistosoma mansoni infection in a five-year randomized trial of praziquantel preventative chemotherapy strategies [published correction appears in J Infect Dis. 2018 May 15;217(10):1672.]. J Infect Dis. 2017;216(11):1425–1433. doi: 10.1093/infdis/jix496 28968877 PMC5913648

[14] Kittur N, Binder S, Campbell CH, King CH, Kinung’hi S, Olsen A, et al. Defining persistent hotspots: areas that fail to decrease meaningfully in prevalence after multiple years of mass drug administration with praziquantel for control of schistosomiasis. Am J Trop Med Hyg. 2017;97(6):1810–1817. doi: 10.4269/ajtmh.17-0368 29016344 PMC5805060

[15] Fenwick A, Webster JP, Bosque-Oliva E, Blair L., Fleming FM, Zhang Y, et al. The Schistosomiasis Control Initiative (SCI): rationale, development and implementation from 2002–2008. Parasitology. 2009;136(13):1719–1730. doi: 10.1017/S0031182009990400 19631008

[16] Fenwick A. New initiatives against Africa’s worms. Trans R Soc Trop Med Hyg. 2006;100(3):200–207. doi: 10.1016/j.trstmh.2005.03.014 16343572

[17] Clements AC, Lwambo NJ, Blair L, Nyandindi U, Kaatano G, Kinung’hi S, et al. Bayesian spatial analysis and disease mapping: tools to enhance planning and implementation of a schistosomiasis control programme in Tanzania. Trop Med Int Health. 2006;11(4):490–503. doi: 10.1111/j.1365-3156.2006.01594.x 16553932 PMC2202922

[18] Fleming FM, Fenwick A, Tukahebwa EM, Lubanga RG, Namwangye H, Zaramba S, et al. Process evaluation of schistosomiasis control in Uganda, 2003 to 2006: perceptions, attitudes and constraints of a national programme. Parasitology. 2009;136(13):1759–1769. doi: 10.1017/S0031182009990709 19695107

[19] French MD, Churcher TS, Gambhir M, Fenwick A, Webster JP, Kabatereine NB, et al. Observed reductions in Schistosoma mansoni transmission from large-scale administration of praziquantel in Uganda: a mathematical modelling study. PLoS Negl Trop Dis. 2010;4(11):e897. doi: 10.1371/journal.pntd.0000897 21124888 PMC2990705

[20] Ouedraogo H, Drabo F, Zongo D, Bagayan M, Bamba I, Pima T, et al. Schistosomiasis in school-age children in Burkina Faso after a decade of preventive chemotherapy. Bull World Health Organ. 2016;94(1):37–45. doi: 10.2471/BLT.15.161885 26769995 PMC4709800

[21] World Health Organization. Summary of global update on implementation of preventive chemotherapy against neglected tropical diseases in 2019. Weekly epidemiological record. 2020;39(95):469–74.

[22] Chami GF, Kontoleon AA, Bulte E, Fenwick A, Kabatereine NB, Tukahebwa EM, et al. Community-directed mass drug administration is undermined by status seeking in friendship networks and inadequate trust in health advice networks. Soc Sci Med. 2017;183:37–47. doi: 10.1016/j.socscimed.2017.04.009 28458073 PMC5446315

[23] Mwanga JR, Kinung’hi SM, Mosha J, Angelo T, Maganga J, Campbell CH. Village response to mass drug administration for schistosomiasis in Mwanza region, northwestern Tanzania: are we missing socioeconomic, cultural, and political dimensions? Am J Trop Med Hyg. 2020;103(5):1969–1977. doi: 10.4269/ajtmh.19-0843 32901610 PMC7646777

[24] Parker M, Allen T, Hastings J. Resisting control of neglected tropical diseases: dilemmas in the mass treatment of schistosomiasis and soil-transmitted helminths in north-west Uganda. J Biosoc Sci. 2008;40(2):161–181. doi: 10.1017/S0021932007002301 17761005

[25] Kabatereine NB, Fleming FM, Nyandindi U, Mwanza JC, Blair L. The control of schistosomiasis and soil-transmitted helminths in East Africa. Trends Parasitol. 2006;22(7):332–339. doi: 10.1016/j.pt.2006.05.001 16713357

[26] Schensul SL, Schensul JJ, LeCompte MD. Initiating ethnographic research: A mixed methods approach. United Kingdom: AltaMira Press; 2012.

[27] Uganda Investment Authority. Hoima District Investment Profile. Kampala: Uganda Investment Authority; 2021.

[28] Uganda Bureau of Statistics. The National Population and Housing Census 2014 –Area Specific Profile Series. Kampala, Uganda; 2017.

[29] Guest G, Bunce A, Johnson L. How many interviews are enough? An experiment with data saturation and variability. Field methods. 2006;18(1):59–82.

[30] Uganda Bureau of Statistics. The national population and housing census 2014-main report. Kampala; 2016.

[31] Burns A. Collaborative action research for English language teachers: Cambridge University Press; 1999.

[32] Dawson S, Manderson L, Tallo VLA. manual for the use of focus groups. Boston: International Nutrition Foundation for Developing Countries. 1993.

[33] Creswell JW. Qualitative inquiry and research design: Choosing among five approaches. Thousand Oaks, CA: Sage; 2013.

[34] Armstrong D, Gosling A., Weinman J, Marteau T. The place of inter-rater reliability in qualitative research: An empirical study. Sociology. 1997;31(3):597–606.

[35] Creswell JW. Research design: Qualitative, quantitative, and mixed methods approaches: Sage publications; 2017.

[36] Parker M, Allen T. Does mass drug administration for the integrated treatment of neglected tropical diseases really work? Assessing evidence for the control of schistosomiasis and soil-transmitted helminths in Uganda. Health Res Policy Syst. 2011;9:3. doi: 10.1186/1478-4505-9-3 21211001 PMC3024987

[37] Adriko M, Faust CL, Carruthers LV, Moses A, Tukahebwa EM, Lamberton PHL. Low praziquantel treatment coverage for *Schistosoma mansoni* in Mayuge District, Uganda, due to the absence of treatment opportunities, rather than systematic non-compliance. Trop Med Infect Dis. 2018;3(4):111. doi: 10.3390/tropicalmed3040111 30297642 PMC6306755

[38] Corley AG, Thornton CP, Glass NE. The role of nurses and community health workers in confronting neglected tropical diseases in sub-Saharan Africa: a systematic review. PLoS Negl Trop Dis. 2016;10(9):e0004914. doi: 10.1371/journal.pntd.0004914 27631980 PMC5025105

[39] Krentel A, Gyapong M, Mallya S, Boadu NY, Amuyunzu-Nyamongo M, Stephens M, et al. Review of the factors influencing the motivation of community drug distributors towards the control and elimination of neglected tropical diseases (NTDs). PLoS Negl Trop Dis. 2017;11(12):e0006065. doi: 10.1371/journal.pntd.0006065 29211746 PMC5718409

[40] Fleming FM, Matovu F, Hansen KS, Webster JP. A mixed methods approach to evaluating community drug distributor performance in the control of neglected tropical diseases. Parasit Vectors. 2016;9(1):345. doi: 10.1186/s13071-016-1606-2 27305942 PMC4910194

[41] Basinga P, Gertler PJ, Binagwaho A, Soucat AL, Sturdy J, Vermeersch CM. Effect on maternal and child health services in Rwanda of payment to primary health-care providers for performance: an impact evaluation. Lancet. 2011;377(9775):1421–1428. doi: 10.1016/S0140-6736(11)60177-3 21515164

[42] Leonard KL, Masata MC. Professionalism and the know-do gap: Exploring intrinsic motivation among health workers in Tanzania. Health Econ. 2010;19(12):1461–1477. doi: 10.1002/hec.1564 19960481

[43] Anyolitho MK, Nyakato VN, Huyse T, Poels K, Masquillier C. Health-seeking behaviour regarding schistosomiasis treatment in the absence of a mass drug administration (MDA) program: the case of endemic communities along Lake Albert in Western Uganda. BMC Public Health. 2023;23(1):1072. doi: 10.1186/s12889-023-16020-z 37277773 PMC10240754

[44] Mujumbusi L, Nalwadda E, Ssali A, Pickering L, Seeley J, Meginnis K, et al. Understanding perceptions of schistosomiasis and its control among highly endemic lakeshore communities in Mayuge, Uganda. PLoS Negl Trop Dis. 2023;17(1):e0010687. doi: 10.1371/journal.pntd.0010687 36656869 PMC9888691

[45] Burnim M, Ivy JA, King CH. Systematic review of community-based, school-based, and combined delivery modes for reaching school-aged children in mass drug administration programs for schistosomiasis. PLoS Negl Trop Dis. 2017;11(10):e0006043. doi: 10.1371/journal.pntd.0006043 29077723 PMC5678727

[46] Kabatereine N, Fleming F, Thuo W, Tinkitina B, Tukahebwa EM, Fenwick A. Community perceptions, attitude, practices and treatment seeking behaviour for schistosomiasis in L. Victoria islands in Uganda. BMC Res Notes. 2014;7:900. doi: 10.1186/1756-0500-7-900 25495121 PMC4307169

[47] World Health Organization. Crossing the Billion. Preventive chemotherapy for neglected tropical diseases: Lymphatic filariasis, onchocerciasis, schistosomiasis, soil-transmitted helminthiases and trachoma. Geneva: World Health Organization; 2017.

[48] World Health Organization. Preventive chemotherapy in human helminthiasis. Coordinated use of anthelminthic drugs in control interventions: a manual for health professionals and programme managers. Geneva: WHO; 2006.

[49] Hastings J. Rumours, riots and the rejection of mass drug administration for the treatment of schistosomiasis in Morogoro, Tanzania. J Biosoc Sci. 2016;48 Suppl 1:S16–S39. doi: 10.1017/S0021932016000018 27428064

[50] World Health Organization. Safety in administering medicines for neglected tropical diseases. Geneva: WHO; 2021.

[51] Sacolo H, Chimbari M, Kalinda C. Knowledge, attitudes and practices on Schistosomiasis in sub-Saharan Africa: a systematic review. BMC Infect Dis. 2018;18(1):46. Published 2018 Jan 18. doi: 10.1186/s12879-017-2923-6 29347919 PMC5773048

[52] Gao B, Saralamba S., Lubell Y, White L J, Dondorp AM, Aguas R. Determinants of MDA impact and designing MDAs towards malaria elimination. Elife. 2020;9:e51773. doi: 10.7554/eLife.51773 32293559 PMC7185997

[53] Coulibaly JT, Ouattara M, Barda B, Utzinger J, N’Goran EK, Keiser J. A rapid appraisal of factors influencing praziquantel treatment compliance in two communities endemic for schistosomiasis in Côte d’Ivoire. Trop Med Infect Dis. 2018;3(2):69. doi: 10.3390/tropicalmed3020069 30274465 PMC6073597

[54] Torres-Vitolas CA, Trienekens SC, Zaadnoordijk W, Gouvras AN. Behaviour change interventions for the control and elimination of schistosomiasis: a systematic review of evidence from low-and middle-income countries. PLoS Negl Trop Dis. 2023;17(5):e0011315. doi: 10.1371/journal.pntd.0011315 37163556 PMC10202306

[55] Knopp S, Person B, Ame SM, Ali SM, Muhsin J, Juma S, et al. Praziquantel coverage in schools and communities targeted for the elimination of urogenital schistosomiasis in Zanzibar: a cross-sectional survey. Parasit Vectors. 2016;9:5. doi: 10.1186/s13071-015-1244-0 26727915 PMC4700672

