# Supplementary material for: Barriers and facilitators to programmatic mass drug administration in persistent schistosomiasis hotspot communities: An ethnographic study along Lake Albert, midwestern Uganda
Source: PLoS Negl Trop Dis. 2024 Dec 13;18(12):e0012002. doi: 10.1371/journal.pntd.0012002 (PMC11676557; doi:10.1371/journal.pntd.0012002)
Supplement: S1 Table — Provided are the summary statistics for age, gender, educational level, marital status and reported S. mansoni infection status of participants. (DOCX) [file pntd.0012002.s001.docx]

**S1 Table**: **Socio-demographic characteristics of the in-depth interview participants**

| **Age range in years** | **N (%)** |
| --- | --- |
| 18-30 | 26 (50.0) |
| 31-40 | 14 (26.9) |
| 41-50 | 5 (9.6) |
| 51+ | 3 (5.8) |
| Not recorded on transcript | 4 (7.7) |
|  | |
| **Gender** |  |
| Male | 30 (57.7) |
| Female | 21 (40.4) |
| Not recorded on transcript | 1 (1.9) |
|  |  |
| **Education Level** |  |
| None | 1 (1.9) |
| Primary | 16 (30.8) |
| Secondary | 21 (40.4 |
| Tertiary | 1 (1.9) |
| Not recorded on transcript | 13 (25) |
|  |  |
| **Marital Status** |  |
| Single | 12 (23.1) |
| Married | 29 (55.8) |
| Separated | 3 (5.8) |
| Widowed | 1 (1.9) |
| Not recorded on transcript | 7 (13.4) |
|  |  |
| **Reported *S. mansoni* Infection Status** |  |
| Ever had | 38 (73.1) |
| Never had | 6 (11.5) |
| Not recorded on transcript | 8 (15.4) |
