# Supplementary material for: Barriers and facilitators to programmatic mass drug administration in persistent schistosomiasis hotspot communities: An ethnographic study along Lake Albert, midwestern Uganda
Source: PLoS Negl Trop Dis. 2024 Dec 13;18(12):e0012002. doi: 10.1371/journal.pntd.0012002 (PMC11676557; doi:10.1371/journal.pntd.0012002)
Supplement: S2 Table — Provided are the ranges in age, educational status and the gender of the participants for each group. (DOCX) [file pntd.0012002.s002.docx]

**S2 Table:** **Socio-demographic Characteristics of the focus group discussion participants**

| **FGD** | **Site** | **Number of participants** | **Age Range/Category** | **Final Year Education (Range)^*^** | **Gender** |
| --- | --- | --- | --- | --- | --- |
| 1 | Kaiso | 6 | 25-45 | P3 -S1 | Female |
| 2 | Kaiso | 7 | Youths | P7-S4 | Male |
| 3 | Kaiso | 7 | Youths | P3-S1 | Female |
| 4 | Kaiso | 7 | 36-60 | 0-P6 | Female |
| 5 | Buhirigi | 7 | Youths | P4-S3 | Male |
| 6 | Buhirigi | 7 | Youths | 0-S4 | Female |
| 7 | Buhirigi | 7 | 31-50 | 0-P6 | Female |
| 8 | Buhirigi | 7 | 18-45 | P2-S4 | Male |
| 9 | Kaiso | 7 | Youths | P5-S4 | Male |
| 10 | Kaiso | 7 | 31->60 | P5-S4 | Male |
| 11 | Buhirigi | 7 | 18-35 | P1-S2 | Male |
| 12 | Buhirigi | 7 | Youths | P4-S2 | Male |
| 13 | Buhirigi | 7 | 36-55 | P4-S2 | Male |
| 14 | Buhirigi | 7 | 36-55 | 0-P7 | Female |

^*^P = Primary, S = Secondary
